# Exploring Feeding Practices and Food Literacy in Parents with Young Children from Disadvantaged Areas

**DOI:** 10.3390/ijerph18041496

**Published:** 2021-02-04

**Authors:** Jennifer Tartaglia, Michelle McIntosh, Jonine Jancey, Jane Scott, Andrea Begley

**Affiliations:** 1 Foodbank Western Australia, Perth Airport, Perth, WA 6105, Australia; jennyt@foodbankwa.org.au (J.T.); michelle.mcintosh@foodbankwa.org.au (M.M.); 2 School of Public Health, Curtin University, Perth, WA 6102, Australia; J.Jancey@curtin.edu.au (J.J.); Jane.scott@curtin.edu.au (J.S.)

**Keywords:** feeding practices, food literacy, nutrition, focus groups, food parenting practices, self-determination theory, responsive feeding

## Abstract

Early childhood provides an opportunity to optimize growth and development and parents play a fundamental role in forming healthy eating habits in their children. A healthy diet improves quality of life and wellbeing and reduces the risk of chronic disease. The aim of this research was to explore parents’ experiences of feeding 0–5-year-old children and food literacy behaviors. This qualitative study employed a general inductive inquiry approach. Participants were recruited through community-based parenting organizations in disadvantaged areas. Eight focus groups were conducted with 67 parents (92.5% female) living in socially disadvantaged areas within metropolitan Perth of Western Australia. Ten themes emerged from the preliminary analysis and were aligned with domains of relatedness, autonomy, and competence within the self-determination theory. Themes included relatedness (1) feeding is emotional, (2) variations in routine and feeding structures, (3) external influences, autonomy (4) power struggles, (5) it must be quick and easy, (6) lack of strategies for feeding autonomy, competency (7) whatever works, (8) healthy is important but for some unattainable, (9) improvements in food literacy skills, and (10) conflicting information overload. This research informed the development of a food literacy program for parents. Parents faced many challenges when trying to provide healthy food. This research has shown parents would benefit from support to achieve healthy eating practices for their families.

## 1. Introduction

Good nutrition during early childhood has been recognized as a critical indicator for optimal health, growth, socio-emotional, language, cognitive, and motor development, particularly in the first 2000 days from conception to five years [1]. Early eating patterns and flavor preferences developed during childhood can persist into later life; therefore, the period when solid foods are introduced to infants is an important stage in forming healthy eating habits [2,3,4]. According to the *Australian Dietary Guidelines*, children should eat sufficient nutritious foods to grow and develop [5]. The guidelines promote a family-centered approach to healthy eating and physical activity as the best way to manage children’s weight. Infant feeding guidelines have been developed to provide evidence-based, best practice recommendations for feeding children from birth to two years of age [6].

The 2017–2018 National Health Survey found that Australian children are not consuming sufficient amounts of nutritious food required for growth and development [7]. The survey, which reported on children aged two years and older, found that 18.2% of 2–3-year-olds and 30.6% of 4–8-year-olds consumed sugar-sweetened drinks weekly [7]. The survey also found that 81.7% of children aged 2–3 years and 95.9% of children aged 4–8 years did not achieve the recommended daily vegetable intake. The Australian Bureau of Statistics estimates that one in four (24.6%) 2–4-year-olds are overweight or obese [7]. Studies of Australian children aged less than 2 years indicate that the consumption of discretionary foods that are energy dense and nutrient poor begins early in the weaning period and increases markedly in the second year of life [8,9,10]. Additionally, parents and children living with social disadvantage are at greater risk of poor health, including being overweight or obese and, therefore, should be a priority for interventions [11].

Factors influencing eating behaviors are expansive and are impacted by the political, for example, food regulation information; and socio-cultural food environments, for example, food advertising. However, parental food habits and feeding strategies have been found to be the most dominant determinant of feeding behavior [12]. Parents play an integral role in the promotion of their child’s healthy eating behavior. As the gatekeepers of the early feeding environment, they can influence a child’s long-term eating patterns and health outcomes [13]. Research on early childhood feeding practices has explored approaches that result in positive dietary outcomes. At the forefront is the concept of responsive feeding practice, where parents create a supportive environment that values their child’s ability to self-regulate eating and develop autonomy and provide positive responses that are appropriate to their child’s development and competence [14]. More recently, self-determination theory (SDT) has been used to conceptualize the development and motivation for the responsive feeding practices that are necessary for parents and children to internalize healthy food behaviors and values [14]. The SDT is a motivational theoretical framework, comprising three practices: parental positive involvement (“relatedness enhancing”), autonomy support (“autonomy enhancing”), and provision of structure (“competence enhancing”) [15,16,17]. Ideally, child-centered responsive feeding practices that discourage authoritarian-style parenting behaviors, such as pressure to eat through control, coercion, restriction, and rewards, help to foster a child’s autonomy and eating competency [13,18].

Parents’ own food knowledge and skills are essential for providing healthy diets for their children. In Australia, the concept of food literacy describes the “interrelated factors that are required to plan, manage, select, prepare and eat food to meet dietary needs” ([19], p. 54). The birth of children may be the critical life event that provides an opportunity to improve parents’ food literacy, particularly in light of concerns about intergenerational deskilling and/or devaluating of food skills, such as cooking [20]. Involving young children in food-related activities, such as cooking, models and contributes to their eating competence [21,22]. Little is known about how food literacy behaviors relate to parent feeding practices in Australia; however, time scarcity and responsibility divisions are likely to influence how parents operationalize practices [23]. A lack of food literacy is a significant barrier [24], as lower cooking skills have been associated with a higher proportion of ultra-processed foods in main meals [25].

Feeding experiences have been described as a highly complex social practice that is influenced by the wider environment [13]. Across the 0–5-year age group, there are different experiences and challenges for parents and children, reflecting the rapid growth and development in the first year of life when parents make all the feeding decisions. During the physical and cognitive development that occurs between one and five years, children are expected to develop positive attitudes, food acceptance, and regulation. Qualitative research of the early feeding environment can capture the lived experience of feeding and how parents make sense of this reality through their practices. A recent thematic synthesis of 73 qualitative studies of parents’ attitudes, beliefs, and perceptions regarding feeding in the first year of life found that family, tradition, and culture, including the social norms within the parent’s environment, had the greatest influence in shaping infant feeding behaviors. For example, when to begin complementary feeding and which foods to offer first [26].

In the limited number of Australian qualitative studies, parents of children aged up to five years have reported feeding as a challenging period where they have encountered many barriers [27,28,29,30,31]. Parents consistently report juggling food cost, quality, availability, and marketing influences with their individual circumstances, such as beliefs, family norms, knowledge, skills, and time [27,30,31,32]. There is evidence that parents have nutritional knowledge but struggle to implement it due to factors, such as inconsistent advice and information [33]; trying to avoid conflict; and lacking strategies to overcome the barriers and frustrations they have incurred, such as children refusing the foods offered and managing different food preferences [30]. The Melbourne Infant Feeding, Activity and Nutrition Trial study found that barriers, such as a lack of time, often led parents to make dietary decisions based on what was easy and practical rather than what was healthy [29]. These challenges result in parental tiredness and stress, manifesting in emotionally charged mealtime interactions [34]. Parents describe children as fussy eaters without realizing the normality of adjusting to different food tastes, shapes, colors, and textures [34]. Various theories and frameworks have been applied to these findings to explain parent feeding practices, including ecological systems theory [31]; Capability, Opportunity, Motivation-Behavior theory [29,32]; the PRECEDE–PROCEED framework [27]; and social constructionism [33]. Further investigation of parent feeding practices and food literacy behaviors will assist in the design of education programs to support parents with 0–5-year-olds. This research aimed to explore parents’ feeding practices and food literacy. The objectives were to assess challenges with feeding and strategies used by parents; identify barriers to food planning, selection, and preparation; and explain concepts for nutrition education to inform the development of a food literacy program for parents.

## 2. Materials and Methods

This study used a qualitative methodological approach with a general inductive inquiry [34]. A general inductive inquiry is where interpretations are made from the raw data, without prior assumptions or theories, to build concepts or themes as analysis takes place. Focus groups were chosen as they provide interaction between participants to explore ideas and values and provide a deeper understanding of how attitudes and factors influence the topic [35,36]. Focus groups enable researchers to explore how social or external concepts, such as child feeding recommendations, shape feeding and food literacy behaviors. Curtin University’s Human Research Ethics Committee approved the research (HRE2019-0167-03).

### 2.1. Script Development and Testing

A structured discussion guide (see Supplementary Materials) was developed after reviewing the literature to establish content validity [33,37,38] and to ensure alignment with the objectives of the research. Development of the guide followed the methods used by Krueger and Casey [39]. Face validity was confirmed through interviews with stakeholders from organizations that provide parent-focused services. The first focus group was used as a pilot test, and minor amendments were subsequently made to the wording of questions. A questionnaire collected demographic information relating to sex, age, number and age of children, family role, household composition, level of education, employment status, postcode, being born in Australia, having English as their first language, and identifying as Aboriginal and/or Torres Strait Islander. Four additional validated questions on child feeding and food literacy confidence and behaviors were included to provide context [40]. For each question, participants were asked how often they had undertaken the behavior (offered new foods to your children, eaten a meal with your children, cooked meals at home) or felt confident cooking a variety of meals, in the last month.

### 2.2. Participant Recruitment

Purposeful and snowball sampling was used to recruit parents and carers of at least one child aged 0–5 years. Recruitment focused on low socio-economic-status parents and carers (grandparents or legal guardians) living in metropolitan Perth. The Socio-Economic Indexes for Areas (SEIFA) was used as a proxy measure of socio-economic status. SEIFA is a suite of four indexes developed from a set of socio-economic factors collected from Australian Census data, which ranks geographic areas based on their relative advantage and disadvantage [41]. To access the target group, parent-focused organizations located in socially disadvantaged areas (deciles 1–4) were contacted via email and telephone, and provided with information and advertising material to recruit participants. Five organizations were approached, and all agreed to recruit participants. Organizational staff then recruited interested parents and carers using flyers advertising the focus groups within their centers. Parents/carers provided their names and contact details on a sign-up sheet that was then forwarded to the research team. The research team was not able to follow-up participants who did not attend on the day.

### 2.3. Data Collection

Focus groups were conducted during May and June 2019, at the parent-focused organization as they were familiar places for participants. All of these organizations were established to provide access to child health services, such as child health nurses, and to support parents through parenting programs and social activities (playgroups). Focus groups were conducted by an experienced facilitator and dietitian (A.B.) and nutritionist (J.T.). A third researcher attended to take notes and monitor recording equipment and time, nutritionist (M.M.). Parents were allocated in different groups depending on the youngest child’s age, under two years or between two and five years, reflecting the different stages of growth and development. Four focus groups of between 8 and 12 participants were conducted within each age group (between 64 and 96 participants in total), based on estimations of saturation in the literature [42]. Focus groups ran for approximately one hour and were audio-recorded with participants’ consent. Crèche facilities were provided, where possible, to support participation, and participants received a $20 voucher as reimbursement for their time.

### 2.4. Data Analysis

Responses to demographic and food literacy practice questions were entered into an Excel spreadsheet. Postcodes were converted into SEIFA deciles using data from the 2016 Census of Population and Housing. Postcodes in SEIFA deciles 1–4 were calculated as low, 5–7 as middle, and 8–10 as high socio-economic status. Focus groups were conducted until saturation of ideas was reached [43]. Moderator debriefing with the three researchers occurred directly after each focus group. Audio recordings were transcribed verbatim by a professional service and were managed for analysis using NVivo^®^12 Pro software (QSR International, Melbourne, Victoria, Australia,). The two primary researchers each made notes of emerging ideas after listening to the audio recording. Concurrent data collection and analysis was used with an inductive thematic saturation model as the primary analysis [44]. This saturation model is the extent to which there is non-emergence of new themes and theoretical insights [43]. The phases of the thematic analysis involved familiarization with the data; generating initial codes; searching and reviewing themes; defining and naming themes; and, finally, producing alignment with the research question and selecting representative quotations [44]. Secondary analysis was then applied to the emergent themes, with the application of a theoretical lens to explain and link themes for infant and child feeding. The themes were aligned with constructs of the SDT: relatedness, autonomy, and competence [17]. The quality of all phases of the research was assessed against the Consolidated Criteria for Reporting Qualitative Research checklist to ensure rigor had been achieved when reporting the findings [45].

## 3. Results

Eight focus groups were conducted, involving 67 of the 87 eligible parents and carers (77% response rate). For the purpose of reporting results, all focus group participants who were the primary managers of their child’s dietary intake have been described as parents.

### 3.1. Demographic Characteristics

The characteristics of the participants are reported in Table 1. Participants included parents, grandparents, or guardians of children aged 0–5 years. The majority of participants were female parents (92.5%) aged 26–35 (median age of 34 years), and most families had two or fewer children (77.6%). Of the children aged five and under, 59.4% were aged two years or less, and 40.5% were aged 3–5 years. At the time of the focus groups, just under half of the participants were not in paid employment and reported their roles as house duties or were on maternity leave, and one-quarter were unable to work or were unemployed. Just over half of the participants (57.6%) were living in postcodes with a low SEIFA index, were born outside Australia (56.7%), and spoke English as their first language (59.7%).

### 3.2. Frequency of Food Literacy Practices

New foods were offered to their children “sometimes” (42.4%) or “most of the time” (41%) (see Table 2). Fifty-nine participants reported that they had eaten a meal with their child either “most of the time” or “always” (89.46%). All participants reported that they had cooked meals at home at least sometimes, with almost half of respondents indicating they always cooked meals at home (44.7%). Two-thirds of participants felt confident cooking a variety of meals “most of the time” or “always” (68.2%).

### 3.3. Thematic Analysis

**Figure 1:**
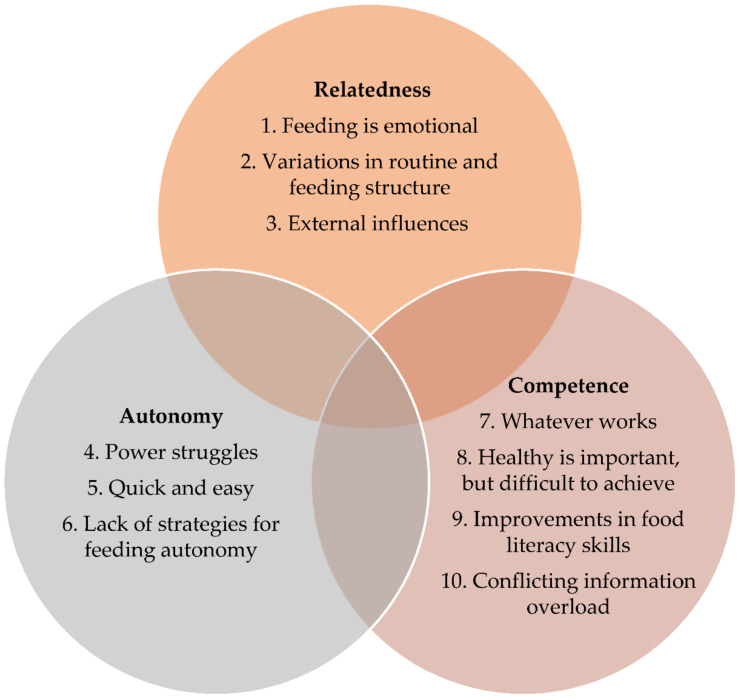
Categorization of themes aligned to self-determination theory (SDT) [17].

The primary analysis revealed 10 themes. These themes were overlayed with the SDT constructs (see Figure 1).

#### 3.3.1. Relatedness Themes

##### Feeding Is Emotional

The experience of feeding children induced a range of emotions in the participants, such as stress, difficulty, worry, and frustration. Participants suggested that factors, such as the anxiety and pressure of feeding, and knowing what to feed children, induced these emotional responses:

*I find that … when [partner’s name] gets home from work, I’m physically, emotionally drained. I seem to hit a barrier when [child’s name] doesn’t eat at night time … last night he literally sat there and screamed … I was in tears as, well, you know, because I said, if he fusses during the day I can deal with it but come night time I am literally physically exhausted ‘cause I work every day as well*.

(FG 1)

Parents experienced anxiety when they perceived their child was not eating well. At the extreme, this anxiety manifested in controlling behaviors, such as weighing food to determine the amount of food eaten. One parent described their own weighing practices, while another described this practice generally:

*I’ve heard of parents that have weighed their food, like, before they’ve given it to the child and weighed everything off the floor after and gone “they’ve eaten bugger all” but it looks like they’ve eaten a decent amount*.

(FG 1)

Frustration was expressed when offering new foods to children. Parents spoke of giving up quickly when foods were rejected and returning to tried-and-tested foods. There was a great deal of discussion about children being fussy eaters because they changed what they liked and disliked. Children eating the food they wanted resulted in conflict and disappointment. They reported being annoyed when children rejected new foods, particularly when they had spent a long time preparing and/or cooking the foods. There was a perception that children’s food preferences became fussier as they grew, as they ate less food and variety, and parents lost control of their child’s feeding:

*Like, I tried grapes with him and no, he just spit it out, so … So, I go back to what he wants, like banana and apples. So at least I know he had one serve of fruit, like, for that day*.

(FG 7)

Many parents spoke of preparing several meals for members of the family to cater for differing preferences to ensure the children ate and to avoid negative food responses:

*I, most of the time, make, like, four different meals but that doesn’t bother me … I grew up in the sense of you have to eat what’s on your plate and [to] the point where you’re sitting there in tears crying and not eating your dinner and it’s horrible. So, I cook what I know they eat, I’m not going to force them to eat anything different*.

(FG 4)

##### Variations in Routine and Feeding Structure

Variation existed in how feeding occurred. Parents spoke about developing feeding routines and structure, and how, before having children, preparing, and eating meals was much more flexible and less planned. Setting a positive example for their children for example, through the social connection of eating together, was important to many parents. However, it was apparent that parents did not always eat with their children, and at times there was little structure or routine around feeding:

*And we will sit down, probably, two to three nights a week, we’ll be able to, yeah, sit down and have dinner with them if I get home early enough to actually have it cooked … Otherwise, yeah, like last night, they had baked beans and toast night for the kids and we had something afterwards as well once they went down to bed*.

(FG 6)

Time was reported as a significant barrier to being more involved with their child’s feeding and providing routine and structure. Cooking for and feeding children needed to be fast, as there were numerous other demands on parents’ time. Parents described waiting until their child was in bed so they could eat their own evening meal, alone or with a partner, so they could sit in peace and be more relaxed:

*I’m a single parent of the two babies … because their dad moved away a year ago, so I just do it from scratch … I don’t eat with the babies, so my time’s very, very limited to getting up and down and catering for them for drinks and all that kind of stuff*.

(FG 2)

##### External Influences

External factors negatively influenced what their children were eating and created tension in the parent–child relationship. Participants perceived there to be a great deal more unhealthy food available now in supermarkets and when eating out, compared to when the parents were children. Parents reported that unhealthy foods were targeted towards young children:

*Too much of [that] sugar[y] stuff is around, yeah, too much cake and biscuits and all this unhealthy stuff out there. Some days I even get so overwhelmed, like, how to stop them from asking when we go to shops or whatever, it’s like, it’s just too much. I think the government should do something about it*.

(FG 6)

Parents found external influences, including judgement from others, disruptive and frustrating. Parents were also critical of other parents’ food choices, particularly how they influenced the types of food their own children started requesting:

*She’s four; she just started kindy. So, like, at home she’ll eat like vegetables and everything but then when I send them to school, it’s the exact same lunch she eats but she won’t touch it because the other kids have chips*.

(FG 8)

#### 3.3.2. Autonomy Themes

##### Power Struggles

Many parents intended to feed healthy foods to their children, but when children became upset, rejected foods, or demanded alternatives, parents gave in to their child’s demands so they could avoid conflict. Children were often making the decisions about what to eat, and parents allowed this to avert power struggles. This produced some anxiety in parents, but seeing their children eat was more important than coercing children to eat; that is, eating something was better than eating nothing:

*No, no, I normally only give him his dessert and generally after he eats. But because yesterday he just refused to eat anything, I thought I’m at a point where … [I gave into his demands and fed him a dessert]. Well, he ate it. Something was better than nothing*.

(FG 1)

Parents used a range of strategies to reduce power struggles, including feeding children in the bath or distracting them with technology, such as television or iPads:

*I’ve got to distract her so she’s not thinking about her eating, to look at the birds outside or to do whatever. Do you know what I mean? She needs a distraction*.

(FG 5)

For others, there was a “take it or leave it” attitude from families who enforced feeding rules. For example, one parent spoke about trying to enforce their rule of not allowing their child to leave the table until the food was eaten.

*Once we’ve [parents] said no to that that he doesn’t get anything else. So, he’ll just have a meltdown if he wants something completely different. When we say no, there’s nothing after that, and he’ll say no I want this, I want that and I’ll say no, this is what’s on offer, you know after that there’s nothing*.

(FG 6)

Variation was apparent in parents’ level of involvement in feeding their children. Many parents did not allow children to feed themselves because they felt it was their job as the parent to ensure their child ate enough food. The mess created by the child was a barrier to children feeding themselves. Other parents encouraged more autonomy with their child’s self-feeding, such as sitting toddlers in high chairs to eat independently, and baby-led weaning, where infants start on solid foods by feeding themselves:

*Yeah, I don’t think I’ve fed my kids since, like, I don’t think [child’s name] was, like, eight months old when I stopped feeding him. As soon as he could sit up, … I sort of skipped purees. So, as soon as he could hold things and eat, he just ate. Yeah, they just, they feed themselves*.

(FG 4)

##### Quick and Easy

Feeding children later in the day needed to be quick and easy and was often considered a task that needed to be ticked off. Children frequently ate dinner earlier than their parents. Time was a barrier to the preparation and eating of nutritious meals. Some parents discussed how commercially prepared baby food, such as that available in small pouches, was an ideal way of feeding younger children, as they were able to be consumed quickly and without any mess:

*You’re spending all this time in the kitchen, like, I might spend hours pureeing food and, if she’s not particularly interested, and that’s hours I could have spent doing something else with her or cleaning or whatever, you know what I mean*?

(FG 8)

The goal was not to spend too much time in the kitchen, preparing meals and cleaning up. Parents described the ideal meal as fast, convenient, and a “quick fix” (FG 8):

*But you know the simmer sauce, you get one for $2 at Coles and Aldi, with steamed rice? I did it better, instant rice, two minutes in the microwave. Yeah, fast, convenient [and] nutritional … like, yeah, bang, 20 min, dinner’s done*.

(FG 1)

The need for quick and easy meals was also evident in how parents would feed their children the evening/main meal separately, earlier in the evening, which was viewed by many parents as a difficult time of day. Working parents identified the demands of coming home from work and having to prepare food as a reason for wanting to feed children quickly and put them to bed, so parents could eat their own evening meal alone or with a partner:

*And that’s one of the big things for me. Like, I know what I want to make them, what I want to feed them but half the time it’s a mad dash to put [together] something that resembles nutrition*.

(FG 6)

##### Lack of Strategies for Feeding Autonomy

There was a notable absence of discussion around building independence and autonomy in children. Strategies to motivate children to eat were more focused “in the moment” rather than on developing a competent and autonomous feeder. The reduction in the amount of food required after the first year of a child’s life, changes in appetite, and developing independence were not well understood. Parents described children as “fussy,” and voiced their frustration at the amount of effort it took to feed their child. Parents did not discuss their child’s independence as an opportunity to move towards their child building autonomy or skills around eating.

*I just tell her, like, eat, you know, two or three mouthfuls or whatever cause I always said you used to eat it. Like, you literally ate it six months ago, like, I don’t understand*.

(FG 4)

The emphasis on keeping things quick and easy were barriers to providing opportunities for children to develop autonomy and competence around feeding. Parents were mostly unwilling to allow their children to help with meal preparation, as involving children was messy and time-consuming. A few parents involved their children in meal preparation tasks, such as chopping, peeling, and spreading foods:

*I don’t mind them helping in the kitchen, but when it’s a rush, like, just get away, no, go away*.

(FG 8)

#### 3.3.3. Competence Themes

##### Whatever Works

Parents were motivated to provide healthy food for their child; however, they described doing “whatever worked” (FG 1) to motivate their children to eat period. A large amount of time and effort was spent encouraging children to eat certain foods by presenting foods in different and appealing ways. For example, providing food that was colorful and was prepared in appealing shapes. Other tactics included hiding or disguising vegetables by pureeing and mixing them into other foods, or adding sauces, cheese, salt, butter, or ghee. Another strategy was to present lots of food options for their children. Many parents reported continually searching on the internet or YouTube to find recipes for new and exciting foods for their children. Often, parents would forgo the priority to feed their children a nutritious meal just to see them eat. For example, commercially prepared baby food or baby food pouches were a fail-safe option but were often associated with guilt or shame as they were perceived as inferior to home-prepared food:

*Yeah, she’ll eat three times a day but, ‘cause she’s so little, if she’s in a bad mood, she doesn’t want to eat … so then instead of eating what I prepared for her or what I want her to eat, it’ll be one of those, you know, Heinz food pouches or some avocado or something that I’ll know she’ll eat as opposed to me being, like, no, let’s try to feed her this because this is what she needs. But if it’s a battle, let’s just feed you*.

(FG 9)

##### Healthy Is Important but Difficult to Achieve

Factors, such as time and cost, dictated what was purchased and cooked. Cooking at home was a way of saving money for some; however, others expressed the view that healthy food was expensive and unattainable on a budget. Conversely, some parents chose takeaway food over home-cooked meals, as it was viewed as a cheaper option that would be eaten with minimal waste compared to homemade food:

*[Buying commercially prepared food is] more convenient … then[sic] trying to, you know, cook it, prepare it, you know, put it in the freezer, let it cool down and then bring it out and freeze it. That’s so much hard work*.

(FG 5)

There was frustration at having spent a long time cooking when children did not eat the food. An extreme response from one parent was that only giving fast food was an easy and fail-safe option. Seeing their child eat was the most important factor, regardless of whether the food was healthy or not:

*Better off going to McDonald’s. Seriously, I feel like that sometimes, when [child’ name] gets the better of me because, you know, it’s like 15 bucks [for] Maccas; it’s 10 bucks to cook a tuna pasta bake. Tuna pasta bake, like, lasts, what, three days? My kids don’t mind it but they won’t all sit down and eat it*.

(FG 1)

Parents who were born overseas described how foods in Australia were different from the foods they had eaten as a child, and what they were accustomed to from their own country. They also expressed that they did not have an understanding of “Aussie” foods, and their knowledge of healthy food was limited.

Although not overtly discussed, there was evidence that some parents were food insecure. After the completion of focus groups, three parents separately approached the researchers to inquire how they could obtain food assistance. There was discussion about how money did not go far and how expensive having children was. Some employed money-saving strategies, such as shopping at discount bulk-shopping stores to look for specials. For a small group of parents, food insecurity was a barrier to providing a range of food choices. The priority for this group was ensuring their children did not go hungry, which reduced the opportunity for food choices and child autonomy around food decisions:

*It becomes difficult when you’ve got, like, 30-odd dollars for the nappies, 30-odd dollars for wipes … if you’re buying the purees and the tins, it just there is [sic] some fortnights I’m spending up to $150 sort of thing on all of that when I can spend 100-odd dollars, 75-odd dollars if I make it myself*.

(FG 3)

##### Improvements in Food Literacy Skills

There was discussion that food literacy skills and confidence had improved since becoming parents. Compared to before having children, parents planned and cooked more meals, and relied less on takeaway foods; they had to learn how to cook and have more healthy food available:

*Oh, it was very easy when I was at work. I wasn’t getting home ‘til eight [or] nine o’clock at night and I would just eat junk food, whatever, chocolate. I’d go to the chippy and I’d get, you know, chips or whatever. So, it’s made me cook. I don’t enjoy cooking at all and I have to do it. I’m not good … mine’s [diet] totally changed ‘cause I used to have a terrible way of eating for myself*.

(FG 8)

Parents spoke of eating more vegetables and increasing efforts to eat healthy foods since having children. Big “shop ups” for fruit and vegetables at markets or food outlets ensured healthy foods were available for the entire week. Parents planned meals and pre-cooked meals for freezing to ensure meals could be easily prepared each day. Many parents also kept commercially prepared baby food in jars or pouches on standby as a backup:

*Yeah. More meal prepping, even like batch cooking, just having it all, ‘cause it’s hard to, sort of, ok, now we’ve got to do a meal or have something on hand*.

(FG 3)

Many parents born overseas cooked at home, and these parents saw cooking as the mother’s role. Value was placed on food as a way of experiencing social connection within families and a focus of celebrations. Parents discussed how, since arriving in Australia, they were keen to learn to cook more “Aussie” foods, often searching the internet to discover how to cook a variety of typically Australian foods.

##### Conflicting Information Overload

Parents obtained feeding information from various sources, including websites, books, social media pages, friends, family, mothers’ groups, social groups, such as playgroups held at parenting organizations, and child health nurses. The amount of information was overwhelming and sometimes conflicting, making it difficult to navigate. Parents reported receiving advice from a health professional to introduce foods and feed children one way, and contradictory advice from others. Some parents relied on information given to them from their own mothers and family members; however, some parents did not have family to ask, so they relied on advice from friends or information from websites:

*Search, yes, if I got confused, just Google or ask grandparents, like, because we’ve come from [a] different country. So, sometimes I’ll ask my parents, sometimes I ask my husband’s parents*.

(FG 4)

Parents had difficulty verbalizing what they wanted from a nutrition education program. After some prompting, the suggested topics included budget-friendly recipes, lunch boxes, quick and easy meals and snacks, help with fussy eaters, and serving sizes for children:

*How you can make tasty food for them that, … with minimal ingredients? … something that goes further … can keep for a while, [is] freezable*.

(FG 3)

*Things we can try … the main challenge is really to get them to eat lots of healthy food and how to do that, really … and also to make sure that they are having the right serving*.

(FG 7)

## 4. Discussion

The findings provide an insight into the feeding experiences of parents from socially disadvantaged areas. It is the first qualitative study to focus feeding and food literacy of Western Australian parents of 0–5-year-olds. Qualitative research plays an important role in understanding facilitators and barriers to healthy eating behaviors. The themes generated from the eight focus groups indicate that parents are motivated to provide nutritious foods but that feeding children under five years presents a number of challenges. This study examined the results through the lens of SDT constructs, which directed the discussion to strategies that enhance parents’ intrinsic motivation to develop autonomous competent eaters. Increasing this type of motivation leads to self-determining feeding practices and improved food literacy behaviors. The SDT application indicates that parents’ intrinsic motivation to provide a nutritious diet for their child was overridden by extrinsic barriers.

### 4.1. Relatedness Enhancing

When children build a sense of relatedness, emotional support, and positive connection to their parents, it can be a powerful facilitator for the adoption of healthy eating habits [17]. The home food environment plays an important role in a child developing competency to self-regulate their eating behaviors. Relatedness enhancing parenting practices, such as nurturing and providing structure, routines, and clear expectations, have been positively associated with healthy dietary intakes [13]. Parents can foster feeding competency and mastery to produce healthy eating behaviors in their child by teaching rules about healthy food (i.e., rules around snack consumption); providing a healthy food environment, including food availability and accessibility and mealtime structure (i.e., regular meals and snack times); and direct modelling (i.e., a parent eating healthy food with their child), where a child learns through observing and imitating behaviors [17]. Through these parenting practices and socialization, children can develop positive internalization of healthy behaviors and values from their parents.

Consistent with other Australian research, our research found that parents experienced a number of emotional responses to feeding children, particularly when they considered their children to be fussy eaters. Fussy eating has been described to result in anxiety, frustration, and stress in parents [30]. Research has shown that a lack or absence of positive feeding experiences, positive parental involvement, warmth and support for their child, as well as the use of unhealthy food to regulate a child’s emotions, can negatively impact a child’s ability to develop a positive connection, or relatedness, with their parents [17]. Poor connections can reduce parents’ motivation to achieve nutrition-oriented goals, such as encouraging their child to eat nutritious foods, and psychosocial goals, such as wanting their child to feel secure, well fed, and safe [38].

Fussy eating is reported as a common behavior in early childhood [28]. Although research has indicated that it is unlikely to cause any permanent harm to a child’s development, it does cause stress for parents and can have a negative impact on family relationships. Strategies and advice for parents to prevent or improve fussy eating include repeated exposures to unfamiliar foods; parental modelling of eating fruit, vegetables, and unfamiliar foods; and the creation of positive social experiences around mealtimes [46].

There were a range of variations in routines and structures around feeding reported by parents in this research. Parents intended to create positive structures and routines but were often challenged by time, cost, competing priorities, and other external influences, such as marketing and peer pressure. This is consistent with research that has shown the variety of external factors that can influence parents’ feeding practices [16,30,38,47,48]. Education and support that address family and cultural priorities, and that empower parents by providing strategies and building confidence to overcome external influences while also preventing or addressing internalized feelings of shame or guilt can be beneficial. Parents who are equipped with techniques or strategies to plan for and manage external influences and the different stages of a child’s development can minimize the use of unstructured and coercive food parenting practices [49].

### 4.2. Autonomy Enhancing

Parental approaches to feeding can vary widely [18]. This study identified feeding behaviors that both built and thwarted the child’s autonomy. To avoid conflict with their children, parents provided unhealthy food choices driven by the wants of the child, gave in to their child’s demands, and/or were restrictive and rigid about the foods that were provided. For example, just under half of the participants in this study indicated they infrequently introduced new foods to their child. Limited time is a pressure that can result in authoritarian feeding practices that override strategies to develop autonomy [18,37,38,39,40,41,42,43,44,45,46,47,48,49,50].

Excessive food restriction, pressure to eat, and control by parents can have unintended negative consequences on children’s eating behaviors and have been associated with an increase in children’s body mass index [50]. Restrictive feeding practices can result in increased snacking in children aged 2–18 years [51]. Responsive feeding is a fundamental philosophy underpinning the Satter Eating Competence Model [52] and Division of Responsibility [53] feeding strategies, both of which focus on building a child’s self-competency and autonomy. Allowing children to intuitively eat enough food, rather than parents controlling or restricting food, helps them to develop internal regulation skills [54]. Furthermore, autonomy can be strengthened if children are involved in decision-making through a guided choice in planning and preparing meals. Parents can foster autonomy through nutrition education, praise, and applying negotiation in their child’s food choices [17]. Research has shown that although parents have a desire to provide healthy food, the child’s preferences are a predominant factor in influencing the types of foods served at mealtimes [18,55].

### 4.3. Competency Enhancing

Parents play an important role in the provision of structure in the child’s food environment, such as setting clear rules, modelling healthy eating, and providing healthy food, which helps to develop competence in their child’s ability to self-regulate their eating behaviors. Unfortunately, parents in this study were faced with several barriers, such as lack of time, cost of food, food insecurity, and unfamiliar food, to providing an adequate feeding structure. Parents went to great lengths to convince their children to eat any food but particularly healthy food; however, they often yielded to their child’s demands and forwent their own nutritional goals to placate their child.

Parents were faced with multiple and conflicting sources of information. Despite being readily available, information adds to the anxiety parents face when feeding children [26,33]. Consistent with other research, parents in this study often sought feeding advice from family and followed traditional feeding practices passed on by their own parents. Research has found parents may be more prepared to take advice from family members rather than health professionals; however, this information can be unhelpful or misinformed. Advice from health professionals may not be practical or relevant to either them or their child’s specific needs. Parents own beliefs, values, and knowledge influences which sources of information are selected and how parents distinguish between sources of information [31,32]. Providing parents with the skills to recognize reputable sources of educational materials, such as government guidelines, and in a format that is easy to comprehend is important [31].

There were several barriers that conflicted with parental nominated goals. These included financial restrictions, busy schedules, poor parental eating habits and modelling, and conceding to their child’s food preferences and requests. Parents in this research did not explicitly discuss many of the acknowledged external factors. Previous research has shown environmental factors influence parents’ selection of healthy food for their families, in particular, their ability to navigate the marketing information on food packaging, lack of certainty about packaging information, pressure to meet multiple demands, together with a desire to shop quickly, and the conflict between feeding children well and keeping them happy [56]. Researchers advocate for parent programs that acknowledge and value the relevance of both psychosocial goals, such as wanting to help children feel secure and nutrition goals (providing a nutritious diet) while assisting parents to develop strategies to address tensions between goals [38]. In addition, programs can raise parents’ awareness of the impact of the broader political and socio-cultural environment on food choices.

Some parents stated that they experienced food insecurity, which further exacerbated unhealthy feeding practices. American research with parents of two-year-olds from food-insecure households reported more pressure to eat energy-dense nutrient-poor foods [57] and food insecurity has been found to be a factor in parents encouraging their children (aged six months to five years) to eat everything on their plate due to the uncertainty of food availability. Although, parents from food-insecure households were motivated to provide healthy meals, and had goals to enhance positive family relationships through family meals; barriers, such as time and food costs, influenced these goals, resulting in parents adopting less healthy feeding practices [58]. Parents, particularly those that are food insecure, need assistance to balance dietary goals, and food literacy knowledge, skills, and resources to achieve healthy eating practices [38,57]. Linking parents to food relief services may provide additional support for such families. In addition, providing a free nutrition education/food literacy program with the provision of free child care has been recommended [58].

Through education, parents can be empowered to gain a better understanding of a healthy diet and learn practical ways to form healthy dietary behaviors in their child during the early years [59,60]. Many parents in our study were using food literacy behaviors more frequently and/or experienced improvements in food literacy skills since having children; however, it was evident that further improvements in these skills would assist parents in overcoming the barriers to providing healthy meals for their families. Higher parental confidence in cooking skills and in selecting foods by understanding labels has been associated with lower consumption of ultra-processed foods by children; this also increases healthy eating behaviors, such as sharing cooking skills with children [60,61]. Teaching children about food, in a way that is appropriate for their developmental stage, through activities that provide just enough structure and assistance to help them to learn is the aim.

Although interventions that incorporate cooking skills are widely used in public health nutrition interventions and have resulted in favorable dietary outcomes, such as healthier food choices and other health-related outcomes [60,62], few interventions have investigated the concepts of both food literacy skills and parenting practices to improve health outcomes for families. For parents to achieve healthy dietary outcomes for families with children aged 0 to 5 years, there is a need to improve their food literacy skills and increase knowledge about creating positive feeding environments. Parents need educational support that addresses their personal feeding choices and ideology in a way that is easy to understand, factual, user-friendly, and culturally appropriate [26]. Parents also value social interactions with other parents; this, too, can provide learning opportunities and foster the adoption of healthy feeding practices [32].

### 4.4. Limitations

This research used a number of strategies to ensure rigor or trustworthiness in the findings, including the results are credible and repeatable with the same cohort, and reflexivity and theoretical triangulation to achieve confirmability. In considering the generalizability of results, however, participants were purposively selected from disadvantaged areas within the Perth metropolitan area only; as such, findings may not represent the population of all parents. While areas of disadvantage were chosen as the setting for this study, some participants reported living in a postcode from middle to high socio-economic areas. Participants were mostly female. Focus groups were only conducted during the daytime, which may have restricted some parents, particularly males from attending.

Another limitation is that this research did not explicitly explore the political and socio-cultural environments that impact on food choices but have reported on where focus group participants did raise issues, such as marketing.

## 5. Conclusions

Parents of 0–5-year-olds were found to have motivation and positive intentions regarding their child’s nutrition but were often challenged when trying to provide healthy food using positive feeding practices and food literacy behaviors. This research has shown that parents would benefit from support to achieve healthy eating practices for their families as they struggle with both “what” to feed and “how” to feed. Many parents face a range of barriers and challenges in providing nutritious meals for their families on a daily basis. Parent nutrition-education programs should aim not just to improve parents’ food literacy skills (i.e., planning, management, selection, preparing, and eating healthy food) and knowledge, but also to develop and strengthen parenting practices by enhancing relatedness, autonomy, and competency to achieve healthy outcomes. This research has provided an insight into parents’ experiences of feeding their children aged 0–5 years and will inform the development of a parent food literacy program.

## Figures and Tables

**Table 1:** Characteristics of parents attending focus groups.

Characteristic	Responses	*n*	%
**Sex** **(*n* = 67)**	Female	62	(92.5%)
	Male	5	(7.5%)
**Age** **(*n* = 67)**	<18	1	(1.5%)
	18–25	5	(7.5%)
	26–35	34	(50.7%)
	36–45	22	(32.8%)
	≥46	5	(7.5%)
**Family role** **(*n* = 67)**	Parent	62	(92.5%)
	Grandparent	3	(4.5%)
	Carer/guardian	2	(3.0%)
**Number of children** **(*n* = 67)**	1	27	(40.3%)
	2	25	(37.3%)
	3	9	(13.4%)
	≥4	6	(9.0%)
Age of children * **(*n* = 87)**	<1 year	16	(18.4%)
	1–2 years	28	(32.2%)
	3–5 years	30	(34.5%)
	≥6 years	13	(14.9%)
**Household composition** **(*n* = 67)**	Couple with children	50	(74.6%)
	Single parent with children	12	(17.9%)
	Carer/guardian/grandparent	3	(4.5%)
	Living with extended family	2	(3.0%)
**Education level** **(*n* = 67)**	Certificate or diploma	24	(35.8%)
	Bachelor degree or higher	23	(34.3%)
	Some high school	13	(19.4%)
	Finished high school	7	(10.4%)
**Employment status** **(*n* = 67)**	Not currently working	29	(43.3%)
	Unemployed	14	(20.9%)
	Part-time or casual	13	(19.4%)
	Full-time	5	(7.4%)
	Unable to work/disability	3	(4.5%)
	Maternity leave	2	(3.0%)
	Self-employed	1	(1.5%)
SEIFA ** **(*n* = 66)**	Low (decile 1–4)	38	(57.6%)
	Middle (decile 5–7)	25	(37.9%)
	High (decile 8–10)	3	(4.5%)
**Born in Australia** **(*n* = 67)**	No	38	(56.7%)
	Yes	29	(43.3%)
**English as first language** **(*n* = 67)**	Yes	40	(59.7%)
	No	27	(40.3%)
**Identify as Aboriginal or Torres Strait Islander** **(*n* = 67)**	Yes	15	(22.4%)
	No	52	(77.6%)

* Participants could have more than one child. ** SEIFA derived from postcode using Index of Relative Social-economic Advantage and Disadvantage, 2016 [42].

**Table 2:** Frequency of food literacy practices of focus group participants.

Food Literacy Practice	Response	*n*	%
Offered new foods to your children (*n* = 66)	Never/rarely	4	6.0%
	Sometimes	28	42.4%
	Most of the time	27	41.0%
	Always	7	10.6%
Eaten a meal with your children (*n* = 66)	Never/rarely	3	4.5%
	Sometimes	4	6.1%
	Most of the time	28	42.4%
	Always	31	47.0%
Cooked meals at home (*n* = 67)	Never/rarely	0	0.0%
	Sometimes	4	6.0%
	Most of the time	33	49.3%
	Always	30	44.7%
Felt confident cooking a variety of meals (*n* = 66)	Never/rarely	6	9.1%
	Sometimes	15	22.7%
	Most of the time	20	30.3%
	Always	25	37.9%

## Data Availability

The data are not publicly available due to [insert ethical reasons.

## Supplementary Materials

The following are available online at https://www.mdpi.com/1660-4601/18/4/1496/s1, Table S1: Focus group script.
