# Supplementary material for: Exploring Feeding Practices and Food Literacy in Parents with Young Children from Disadvantaged Areas
Source: Int J Environ Res Public Health. 2021 Feb 4;18(4):1496. doi: 10.3390/ijerph18041496 (PMC7915516; doi:10.3390/ijerph18041496)
Supplement: Supplementary file 1 [file ijerph-18-01496-s001.pdf]

Focus Group Script
Icebreaker –
What is your youngest child's name, age and favourite food.

We are interested in your experiences in feeding in your child/children under the age of 5. You may have older children, however, our focus is only on children 0-5.

1. We are interested in hearing about how decisions are made around meals in your household. *Probe- How is the decision make about what your child eats?*

2. Who is involved in preparation/cooking in your household?

We'd like to get a sense of what meals are like your family, how you and your child/children usually eat. To begin….

3. Think of a time in the day when you might eat with your child (or think about yesterday (dinner last night) when you were feeding your child. *Probe- for context (who, what, where, when, etc.) Identify how parents try to influence their kid's eating, what if they don't finish a meal*

4. When it comes to feeding your child, what comes to mind as being important to you? *Probe- on addressing hunger, waste, finishing plate*

5. Are they any kinds of food-related activities do you like to do with your child?

We hear sometimes about the difficulties parents face when feeding their children.

6. We would like to get a sense of what you think makes it difficult to feed kids- what do you think or hear about the difficulties parents' face then feeding children. *Prob-e How do you deal with this challenge? How or where did you learn to do this? What support or help have you gotten to deal with this challenge? Are there things you wish you could do differently or tried to do differentl*y?

7. Tell us about what things parents can do to help children develop eating habits? *Prompts…focus on behaviours rather than information, routine, rules around mealtimes, role modelling, access to healthy food, where do they access this information to inform their decisions around providing healthy food?*

8. What are some things you have tried to develop healthy eating habits with your child/children? (*Prompts successful not successful*)?

9. How do you use information about feeding kids that you hear about?

DRAFT

We are planning to develop a nutrition program for parents.

10. What would be helpful information to learn in a nutrition program for parents? (What other types of information would you find useful to provide healthy food/meals to your family?) *Prompts…How much and what to feed your child? Recipes? When to introduce solids? Information on allergies Food label reading Lunch boxes, Nutrition and links to child development and growth, Fussy eating, Menu planning & budgeting*

11. Where do you currently get this type of information or help about healthy eating and cooking? (what is difficult about using this information)

12. Is there anything else you think is important to tell us about feeding kids?

DRAFT
